# Production of squalene and fatty acids by *Thraustochytrium* sp. RT2316-16: effects of dissolved oxygen and the medium composition

**DOI:** 10.1186/s40643-025-00937-x

**Published:** 2025-09-16

**Authors:** Paris Paredes, Liset Flores, Mariela Bustamante, Yusuf Chisti, Juan A. Asenjo, Carolina Shene

**Affiliations:** 1 Program of Doctorate in Engineering Science with Specialization in Bioprocess, Universidad de La Frontera, Temuco, 4780000 Chile; 2 Department of Chemical Engineering, Center of Food Biotechnology and Bioseparations, BIOREN, and Centre of Biotechnology and Bioengineering (CeBiB), Universidad de La Frontera, Temuco, 4780000 Chile; 3 Institute of Tropical Aquaculture and Fisheries, Universiti Malaysia Terengganu, Kuala Nerus, Terengganu, 21030 Malaysia; 4 Centre for Biotechnology and Bioengineering (CeBiB), Department of Chemical Engineering and Biotechnology, Universidad de Chile, Beauchef 851, Santiago, 8370459 Chile

**Keywords:** Thraustochytrids, *Thraustochytrium* sp., Microbial squalene, Microbial fatty acids, Eicosapentaenoic acid, Docosahexaenoic acid, Fed-batch fermentation

## Abstract

*Thraustochytrium* sp. RT2316-16 is a versatile marine microbe that produces useful compounds including docosahexaenoic acid (DHA), eicosapentaenoic acid (EPA), carotenoids and coenzyme Q_10_. Unlike the other reported *Thraustochytrium* spp., RT2316-16 is psychrophilic. This report identifies RT2316-16 as a potential source of squalene and assesses squalene accumulation in the biomass under different culture conditions. Furthermore, the effects of dissolved oxygen (DO) concentration on the production of fatty acids are reported for the first time. The squalene content of the biomass depended on the composition of the culture medium and the DO concentration. The accumulation of squalene was promoted when the mass ratio of glucose (or glycerol) relative to organic nitrogen was high and the DO concentration was 20% of air saturation. The highest concentration of squalene in the medium containing glucose was 1.49 ± 0.06 g L^–1^, whereas it was 0.91 ± 0.05 g L^–1^ in the medium containing glycerol. Relatively high concentrations of EPA (224 ± 12 mg L^–1^) and DHA (550 ± 25 mg L^–1^) could be obtained in glucose-containing media with a DO concentration of 20%. A fed-batch operation was found to be suitable for enhancing the concentrations of EPA and DHA. Culture conditions for enhanced production of EPA, DHA and squalene by RT2316-16 are reported. Compared to other thraustochytrids, RT2316-16 was shown to be an excellent producer of squalene.

**Supplementary Information:**

The online version contains supplementary material available at 10.1186/s40643-025-00937-x.

## Graphical Abstract

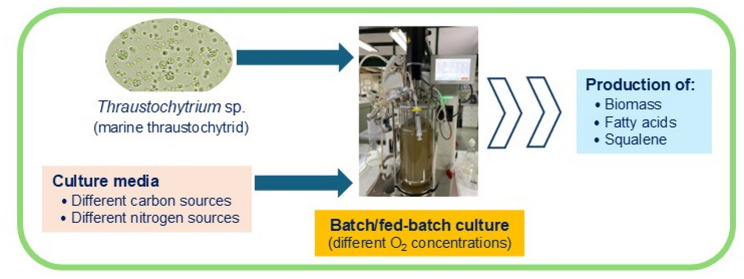

## Introduction

This study focused on the production of squalene and fatty acids by the marine psychrophilic thraustochytrid *Thraustochytrium* sp. RT2316-16. How the culture conditions, especially the concentration of dissolved oxygen, affect the production of squalene and fatty acids in this obligate aerobe is unknown. Squalene (SQ, C_30_H_50_) is a triterpenoid with important applications in cosmetics and pharmaceuticals. Consumption of squalene lowers the risk of heart disease (Aguilera et al. 2005), lowers blood cholesterol (Lou-Bonafonte et al. 2018) and imparts other health benefits (Brown et al. 2019; Güneş 2013; Ronco and De Stéfani 2013). Similarly, consumption of polyunsaturated fatty acids (PUFA) such as eicosapentaenoic acid (EPA, 20:5(*n*-3), C_20_H_30_O_2_) and docosahexaenoic acid (DHA, 22:6(*n*-3), C_22_H_32_O_2_) provides numerous health benefits (Kapoor et al. 2021). In addition to the specific useful metabolites produced by thraustochytrids, their whole biomass has emerging applications in feeds used for aquaculture-based production of seafood (Chi et al. 2022; Naylor et al. 2009). In aquaculture feeds, thraustochytrids provide essential nutrients that are not readily available in feeds from terrestrial source (Chi et al. 2022).

Squalene is a metabolic intermediate of the steroid biosynthesis pathways of all animals and plants. In eukaryotes the precursors of squalene (i.e., isopentenyl pyrophosphate (IPP) and dimethylallyl pyrophosphate (DMAPP), are synthesized from acetyl-CoA in the mevalonate pathway. The accumulation of squalene in the cells is determined by the activity of squalene epoxidase (SQE), also known as squalene monooxygenase. As SQE is inhibited by the antifungal compound terbinafine, its accumulation in the cell is enhanced in the presence of terbinafine (Fan et al. 2010). Microbial production of squalene is further reviewed elsewhere (Paramasivan and Mutturi 2022; Shalu et al. 2024; Xu et al. 2016). Production of fatty acids by thraustochytrids has also been reviewed (Chi et al. 2022; Marchan et al. 2018; Menzorov et al. 2024; Morabito et al. 2019; Raghukumar 2008).

Thraustochytrids are obligate aerobic heterotrophs found in many marine coastal waters (Lyu et al. 2020; Marchan et al. 2018; Raghukumar and Damare 2011). Among microorganisms, thraustochytrids are promising producers of squalene. For example, a squalene content as high as 198 mg g^–1^ (1.29 g L^–1^) has been reported in species such as *Aurantiochytrium* sp. 18 W-13a (Kaya et al. 2011). In addition, thraustochytrids are well-known producers of lipids that are especially rich in DHA. The total lipid content of some species approaches 50% of cell dry mass. Saturated fatty acids in thraustochytrids are synthesized through the classical fatty acid synthase (FAS) pathway whereas the polyunsaturated fatty acids such as DHA are synthesized through polyketide-like PUFA synthase (the anaerobic pathway), although some species may use elongases and desaturases (the aerobic pathway) (Morabito et al. 2019). Thraustochytrids require oxygen irrespective of the pathways being used for fatty acid synthesis. The anaerobic pathway is named as such simply because oxygen is not used within this pathway (Chi et al. 2022).

The accumulation of lipids in thraustochytrids is affected by the culture conditions, including the nutritional composition of the culture medium (concentrations and types of carbon and nitrogen sources), the culture age, and the temperature among other factors (Sohedein et al. 2020). For example, in *Thraustochytrium* sp. the squalene content could be elevated by raising the concentration of NaCl from nil to 5 g L^–1^ (Zhang et al. 2021). This phenomenon was ascribed to enhanced oxygen consumption by the cells grown with NaCl resulting in boosted production of the adenosine triphosphate (ATP) needed in reactions of the mevalonate pathway.

Despite the importance of oxygen supply for metabolism of thraustochytrids, and the possibility of synthesis of the polyunsaturated fatty acids via an oxygen-requiring route and oxygen independent route, few studies have systematically examined the combined effects of DO concentration and media composition on thraustochytrid culture. No such studies have been reported for *Thraustochytrium* sp. RT2316-16 despite its ability to accumulate high levels of lipids (20–45% of its dry weight) (Leyton et al. 2022). RT2316-16 is a versatile producer of multiple high-value compounds (carotenoids, lipids containing EPA and DHA (Leyton et al. 2022); the co-enzyme Q_10_ (Flores et al. 2023; Flores and Shene 2024)) under suitable conditions, making it a worthwhile candidate for investigating production improvements. As in other thraustochytrids, composition of the culture medium affects the growth rate and the biomass composition of RT2316-16. Typically, the media used for growing RT2316-16 contain yeast extract as a source of organic nitrogen and glucose or glycerol as sources of carbon. In this work, RT2316-16 was grown in media with different carbon and nitrogen sources, under different controlled concentrations of DO, to assess the effects on biomass growth and production of total lipids, squalene and fatty acids. As an alternative to the relatively expensive yeast extract, a hydrolyzed lupine (*Lupinus albus*) extract was evaluated as a source of organic nitrogen. Therefore, this study aimed to identify how DO concentration and different carbon and nitrogen sources could be used to influence the production of biomass, squalene and fatty acids by RT2316-16. Such studies are important for developing thraustochytrid-based production of high-value lipids.

## Materials and methods

### Microorganism and culture conditions

All experiments used a pure culture of *Thraustochytrium* sp. RT2316-16 (Leyton et al. 2021) and were conducted aseptically. An initial inoculum was prepared by transferring a loopful (5 µL) of a stock culture (maintained at 4 °C) to 100 mL of the M1 medium in a 250 mL Erlenmeyer flask. The medium M1 had the following composition: glucose (Merck, Darmstadt, Germany) 20 g L^–1^, yeast extract (Merck) 6 g L^–1^, and monosodium glutamate (MSG) (Merck) 0.6 g L^–1^. The medium was made using artificial seawater (see Shene et al. (2013) for composition) diluted with distilled water in a volume ratio of 1:1. In addition, the medium contained a vitamin solution V-I (3.6 mL L^–1^), a vitamin solution V-II (3.6 mL L^–1^), and a solution of mineral salts (24 mL L^–1^). The compositions of V-I, V-II and the mineral salts solutions are specified in Section S1 (Supplemental Material). The vitamins and salt solutions were filter sterilized (0.2 μm sterile membrane filter) prior to use. The inoculated medium was incubated (15 ± 1 °C) in an orbital shaker (150 rpm) for 4 days. An aliquot (10 mL) of the resulting culture was used to inoculate 300 mL (500 mL Erlenmeyer flask) of a medium of the same composition that was to be used in the subsequent bioreactor culture. The incubation conditions were as specified above.

The media evaluated in bioreactor cultures were the following: (1) the medium M1 of the above specified composition; (2) the medium M2 (glucose 5 g L^–1^, yeast extract 12 g L^–1^, MSG 1.2 g L^–1^); (3) the medium M3 (glycerol (Merck, Darmstadt, Germany) 20 g L^–1^, yeast extract 6 g L^–1^, MSG 0.6 g L^–1^); and (4) the medium M4 (lupine extract (Table 1) to replace yeast extract and MSG, and glucose 20 g L^–1^).

The bioreactor batch cultures were carried out in a 6 L Minifors 2 stirred vessel (Infors HT, Switzerland) with a working volume of 3.8 L. The inoculum volume was 300 mL. The dissolved oxygen (DO) concentration was controlled at the specified values using a cascade control protocol involving initial control with the stirrer speed (200–500 rpm) and subsequent control with the aeration rate (1–2 L min^–1^). The carbon and nitrogen sources and their concentrations in the four media used in bioreactor cultures with different controlled concentrations of DO are summarized in Table 1. These combinations of nutrients and DO levels were used to evaluate the culture performance in terms of microbial growth and production of the target metabolites (squalene, fatty acids). The effects of two extreme values of DO, a ‘low’ value and a ‘high’ value, were assessed in most media (Table 1), as metabolic reasoning (see *Introduction*) suggested that production of certain lipids in thraustochytrid could be impacted by oxygen supply.

**Table 1:** Compositions of the media and dissolved oxygen (DO) concentrations used in batch and fed-batch cultures in the bioreactor

Medium	Carbon source (g L^−1^)	Nitrogen source^*^ (g L^−1^)	DO (%)
M1	Glucose (20)	YE (6), MSG (0.6)	5 and 20
M2	Glucose (5)	YE (12), MSG (1.2)	10 and 20
M3	Glycerol (20)	YE (6), MSG (0.6)	10 and 20
M4	Glucose (20)	Lupine extract^†^	20

^*^ YE, yeast extract; MSG, monosodium glutamate; ^†^ equivalent to an Or-N concentration of a yeast extract solution with YE = 6 g L^−1^

Cultures were terminated once the DO concentration began to uncontrollably rise to above the setpoint value indicating a lack of oxygen consumption by the culture due to depletion of a growth-limiting nutrient, generally the nitrogen source in the present work. The DO level rose because oxygen was being supplied through uninterrupted aeration and agitation, but there was barely any consumption as growth had ceased because of insufficiency of the necessary nutrient. The incubation temperature was 15 ± 1 °C. Samples (40 mL) were taken aseptically twice a day. The biomass was recovered by centrifugation (2057×*g*, 4 °C, 10 min) and stored at −18 °C for analysis. The culture supernatant was analyzed for the concentrations of the residual carbon and nitrogen sources.

A fed-batch experiment was also carried out. This began as a batch culture using the medium M1. The first feeding (250 mL) used a concentrated solution containing glucose (140 g L^–1^), yeast extract (84 g L^–1^) and MSG (8.4 g L^–1^). This first feeding aimed to prolong the growth phase of the lipid-free biomass. The second feeding (250 mL) comprised of only glucose (140 g L^–1^) and was aimed at inducing lipid synthesis.

### Preparation of the hydrolyzed lupine extract

Lupine flour was purchased from Avelup S.A. (Temuco, Chile). The flour was mixed with NaOH (0.05 M) to obtain a flour concentration of 50 g L^−1^. A bacterial (*Bacillus* sp.) protease solution (product P3111 Sigma-Aldrich, USA) was added (700 µL protease per g of lupine flour; enzyme activity of ~ 17 U g^−1^). After brief mixing, this slurry was incubated (2 h, 50 °C) in an orbital shaker (150 rpm). The suspended solids were removed by filtration (Whatman 40 paper filter; pore size of 8 μm). The pH of the filtrate was adjusted to 7 with 4 M HCl and it was stored at − 20 °C until use. The lupine extract had organic nitrogen (Or-N) concentration equivalent to Or-N in a yeast extract solution with YE concentration of 35 g L^–1^. The M4 medium contained 0.65 L of the lupine extract in a total volume of 3.8 L.

### Analyses

#### Concentration of total biomass, lipid-free biomass and total lipids

The dry mass concentration of biomass (*x*, g L^−1^) was determined gravimetrically. The culture sample (10 mL) was centrifuged (2057×*g*, 10 min) and the cell pellet was dried to constant weight at 60 °C. The biomass comprised of the lipids (i.e., the storage lipids and structural lipids) and the nonlipid components. The lipid portion of the biomass and the nonlipid part are reported separately, with the latter identified as lipid-free biomass (*X*_*LF*_). This distinction is important because the different fractions of the biomass were affected differently by the composition of the culture medium. The concentration of lipid-free biomass (g L^−1^) was calculated using the following equation:

1 $$\:{X}_{LF}=x\left(1-\frac{l}{100}\right)$$

In the above equation, $\:l$ is the mass percentage of total lipids in the dry biomass. The data on the total lipids in the dry biomass are provided in Tables S2–S5 (see Supplementary Material) for the various experiments. The concentration of total lipids (*TL*, g L^−1^) was calculated using the following equation:

2 $$\:TL=\frac{x\:l}{100}$$

#### Total lipids in biomass and the fatty acid composition of the lipids

The total lipids in the biomass were extracted using a published method (Bligh and Dyer 1959). A 50 mg portion of the dried biomass was extracted (1 h, 150 rpm) with 9.5 mL of a solvent mixture of chloroform/methanol/phosphate buffer (50 mM, pH 7.4) in the volume ratio of 5:10:4. The slurry was transferred to a separating funnel containing 2.5 mL of chloroform. After mixing, 2.5 mL of phosphate buffer was added, the contents were mixed briefly and allowed to separate. The chloroform layer was recovered, and the solvent was evaporated at room temperature in a fume hood. The recovered residue (total lipids) was weighed. The extracted lipids were methylated, and the composition of the fatty acid methyl esters (FAMEs) was determined by gas chromatography (Leyton et al. 2021). A gas chromatograph (GC-2010 Plus; Shimadzu, Kyoto, Japan) equipped with a flame ionization detector and a split injector was used. The column (Rtx-2330; 60 m × 0.32 mm × 0.2 μm film thickness; Thames Restek, Saunderton, UK) used was fused silica capillary and the carrier gas was nitrogen. The temperature program was the following: 140 °C for 5 min, subsequent increase to 240 °C at a rate of 3 °C min^‒1^ and maintained at this temperature for 5 min. The temperature of the injector and the detector was 260 °C. A standard FAME Mix (Supelco, Bellfonte, PA, USA) was used to identify the FAMEs.

### Squalene content of biomass

The squalene content was measured in the total lipids as previously reported (Budge and Barry 2019). Serial dilutions of a standard solution of squalene (0 to 32.9 mg mL^–1^; Sigma-Aldrich, St. Louis, MO, USA) were prepared in chloroform. The calibration curve was linear (*R*^2^ = 0.993) in the above specified concentration range.

### Concentrations of glucose and glycerol

Residual glucose concentration in the cell-free culture medium was measured using the 3,5-dinitrosalicylic acid (DNS) method (Miller 1959). The residual concentration of glycerol was measured by high-performance liquid chromatography (HPLC) (Alliance Waters e2695 Separation Module; Waters Inc, Milford, MA, USA). A refractive index detector (Waters Inc., Milford, MA, USA) was used in combination with a Shodex KS-800 (Showa Denko, Tokyo, Japan) column. The column temperature was 80 °C. The mobile phase (deionized water) flow rate was 1 mL min^− 1^.

### Concentration of organic nitrogen

Concentration of organic nitrogen (amino acids) (Or-N) was determined using the *o*-phthalaldehyde (OPA) method (Nielsen et al. 2001). An aliquot (1 mL) of the reagent solution was mixed with 100 µL of the sample and incubated for 1 to 2 min at 25 °C. Afterwards, the spectrophotometric absorbance was measured at 340 nm. A calibration curve made using standard solutions of yeast extract (0–1 g L^–1^) was used to quantify the Or-N in the sample.

### Statistical analysis

All experiments were performed in duplicate. Average values and standard deviations are reported. MATLAB (MathWorks, Inc., Natick, MA, USA) was used to perform one-way analysis of variance (ANOVA) and comparison of the means at the 95% confidence level.

## Results

### Growth and lipid production in batch cultures

**Fig. 1:**
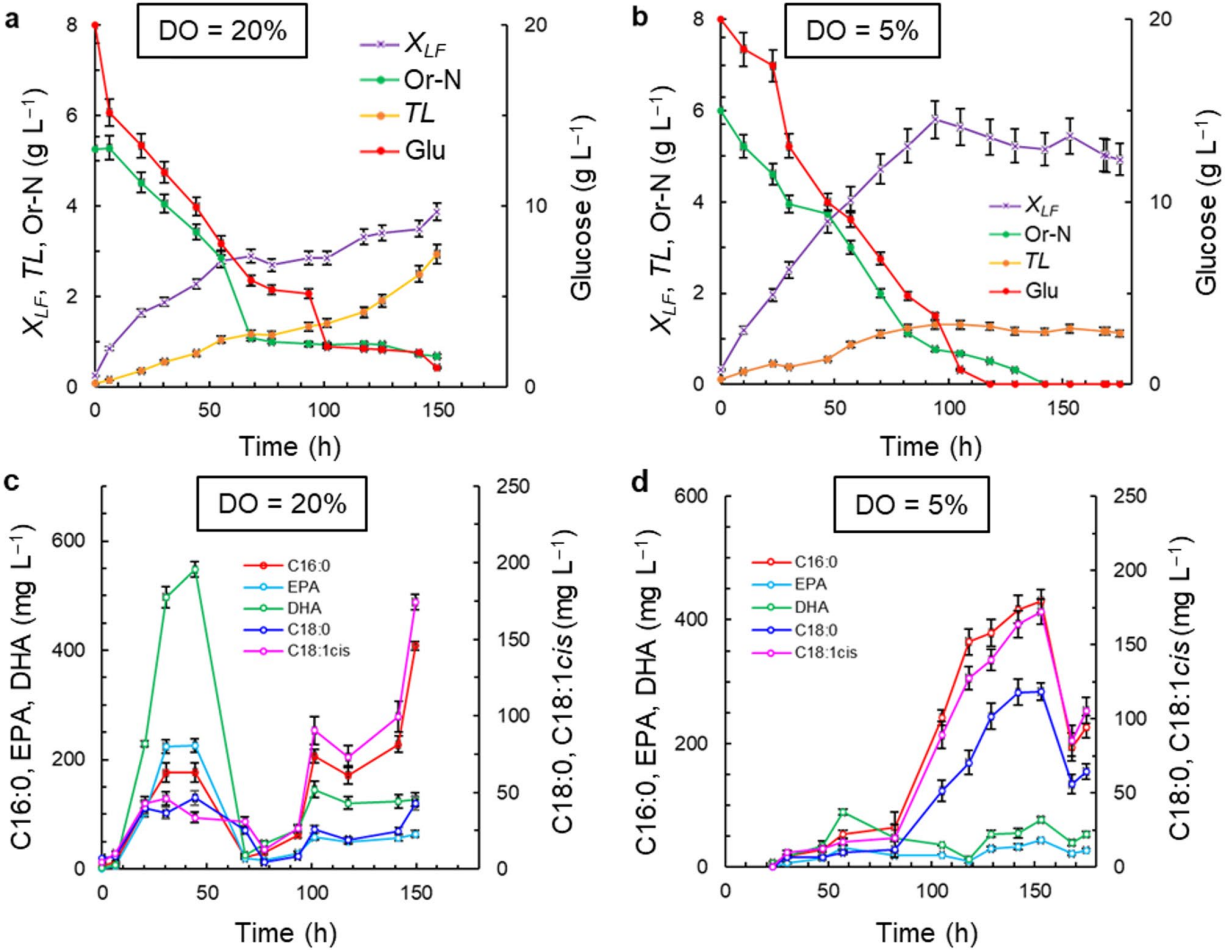
Effects of dissolved oxygen (DO) concentration on the time profiles of the concentrations of lipid-free biomass (*X*_*LF*_), glucose (Glu), organic nitrogen (Or-N), total lipids (*TL*), and specific fatty acids (palmitic acid, C16:0; stearic acid, C18:0; oleic acid, C18:1cis; EPA; and DHA). The medium M1 was used with the following initial composition (g L^–1^): glucose 20; yeast extract 6; and monosodium glutamate 0.6. The medium was made using a 1:1 v v^−1^ mixture of artificial seawater and distilled water

The concentrations of the total lipids and the lipid-free biomass are reported separately because the culture conditions affected them differently. The major components of the lipid-free biomass were proteins, carbohydrates and nucleic acids (Shene et al. 2024). Time profiles of the concentration of the lipid-free biomass, the total lipids, the nutrients, and the key fatty acids (palmitic acid, stearic acid, oleic acid, EPA, and DHA) for the culture in medium M1 (Table 1) at two DO concentrations (DO = 5% and 20%) are shown in Fig. 1. The growth of lipid-free biomass in medium M1 with the high DO level (DO = 20%) followed a diauxic-type pattern (Fig. 1a): during 0–55 h and 101–149 h, the growth was faster than in the intervening period. The rate of consumption of organic nitrogen (Or-N) declined after 77 h, and at termination (150 h) Or-N was not fully consumed (Or-*N* = 0.7 g L^–1^; Fig. 1a). In the medium M1 with the low DO level (DO = 5%), relatively rapid growth of the lipid-free biomass continued to nearly 100 h and a diauxic pattern was not observed (Fig. 1b). The peak concentration of the lipid-free biomass was 5.8 ± 0.4 g L^–1^ at 94 h (Fig. 1b), nearly 50% higher than the peak concentration in the culture with a high DO level (DO = 20%) (Fig. 1a). The estimated growth rate during the 0–94 h was 0.057 g (L h)^−1^. At the low DO level (DO = 5%), glucose was consumed more rapidly than at the high DO level (DO = 20%), and by 119 h it had been fully consumed (Fig. 1b). In contrast, in the high DO level (DO = 20%) culture, the glucose consumption rate appeared to go through multiple distinct phases and by the end of the culture (150 h) a substantial amount (1 g L^–1^) of glucose remained (Fig. 1a).

The DO concentration also affected the total lipids content of the biomass grown in the medium M1 (Table S1; see Supplemental Material) and the production profile of total lipids (Fig. 1a, b). In the low DO culture (DO = 5%), the concentration of total lipids did not increase after around 70 h, and the average final concentration was 1.2 ± 0.5 g L^–1^ (Fig. 1b). In contrast, in the high DO level culture (DO = 20%) the production profile of total lipids had a pattern similar to the growth profile of the lipid-free biomass (Fig. 1a): the concentration of total lipids increased from 0.1 to 1.3 ± 0.1 g L^–1^ between 0 and 93 h and, then, in a second stage (101–149 h), the concentration increased again to finally reach 2.9 ± 0.2 g L^–1^ (Fig. 1a).

The fatty acids in the biomass of RT2316-16 included saturated fatty acids (myristic acid, C14:0; palmitic acid C16:0; and stearic acid C18:0), monounsaturated fatty acids (palmitoleic acid, C16:1; and oleic acid, C18:1) and polyunsaturated fatty acids (PUFA) (linoleic acid, C18:2^D9,12^; g-linolenic acid, C18:3^D6,9,12^; dihomo-γ-linolenic acid, C20:3^D8,11,14^; eicosatrienoic acid, C20:3^D11,14,17^; arachidonic acid, C20:4^D5,8,11,14^; EPA; and DHA). The fatty acids C16:0, C18:0, C18:1, EPA and DHA amounted to 71–91% of the total fatty acids in high DO level (20% DO) culture (Fig. 1c). During the first growth phase of the lipid-free biomass (Fig. 1a), the highest concentrations of C16:0, EPA and DHA were 177 ± 15, 224 ± 12 and 550 ± 25 mg L^–1^, respectively, whereas the concentrations of C18:0 and C18:1 were less than 50 mg L^–1^ each (Fig. 1c). In the second growth phase of the lipid-free biomass (Fig. 1a), the concentrations of C16:0 and C18:1 began to increase to finally reach 525 ± 33 mg L^–1^ for C16:0 and 214 ± 20 mg L^–1^ for C18:1 (Fig. 1c). The average concentrations of EPA and DHA in this period (101–150 h) were 61 ± 9 mg L^–1^ and 129 ± 10 mg L^–1^, respectively. The increase in the concentration of C16:0 was in direct proportion to the increase in the total lipids content of the biomass (Table S1; see Supplemental Material) that took place during the second phase of growth of the lipid-free biomass around 77 h (Fig. 1a). Around the same time, i.e. 77 h, the rate of consumption of Or-N decreased (Fig. 1a).

C16:0 was the main fatty acid (22–56% of total fatty acids) in the biomass grown using the medium M1 with the DO level controlled at 5% of air saturation (Fig. 1d). The concentration of C16:0 began to increase once the growth of lipid-free biomass had ceased (Fig. 1b). The C16:0 concentration reached 430 ± 32 mg L^–1^ after 129 h (Fig. 1d). After glucose and organic nitrogen (Or-N) were depleted (Fig. 1b), the C16:0 concentration decreased substantially (Fig. 1d). The concentrations of C18:0 and C18:1 followed a pattern similar to that of C16:0 (Fig. 1d). The concentrations of EPA and DHA showed a small increase before the concentration of C16:0 increased abruptly (Fig. 1d) although the peak concentrations (43 ± 4 mg L^–1^ for EPA; 88 ± 9 mg L^–1^ for DHA) were smaller than the peak concentration obtained with the high DO level (DO = 20%) (Fig. 1c).

**Fig. 2:**
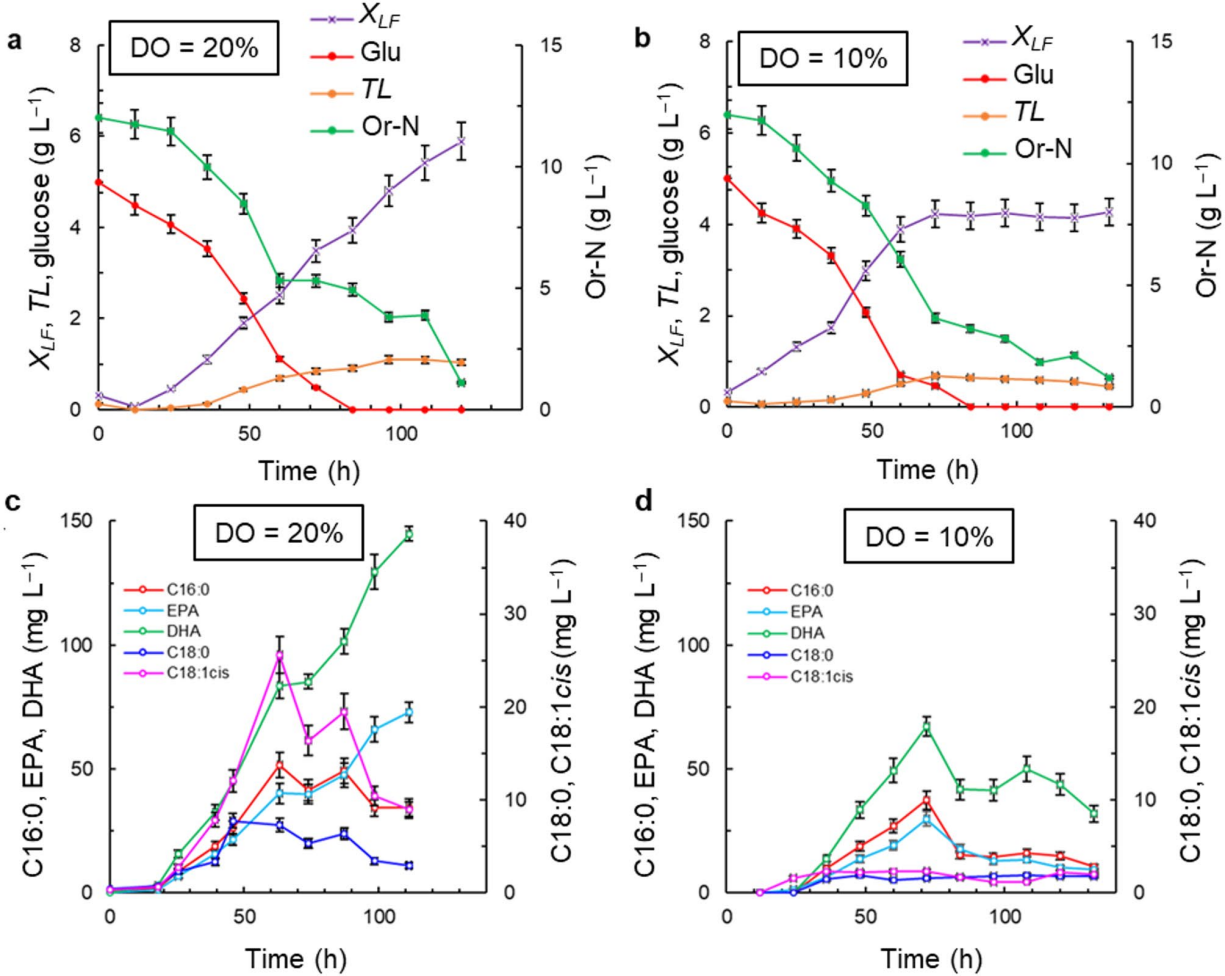
Effects of dissolved oxygen (DO) concentration on the time profiles of concentrations of the lipid-free biomass (*X*_*LF*_), glucose (Glu), organic nitrogen (Or-N), total lipid (*TL*), and specific fatty acids (palmitic acid, C16:0; stearic acid, C18:0; oleic acid, C18:1cis; EPA; and DHA). The medium M2 was used with the following initial composition (g L^–1^): glucose 5; yeast extract 12; and monosodium glutamate 1.2. The medium was made using a 1:1 v v^−1^ mixture of artificial seawater and distilled water

The effects of DO concentrations (DO = 10% and 20%) in medium M2 (Table 1) on RT2316-16 culture profiles are shown in Fig. 2. A DO level of 20% allowed the lipid-free biomass to grow at an average rate equal to 0.057 g (L h)^–1^ (12–120 h) resulting in a final concentration of the lipid-free biomass (5.9 ± 0.4 g L^–1^; Fig. 2a) that was 52% higher than the concentration attained with the medium M1 with the same DO level (Fig. 1a). At the low DO level (DO = 10%), the maximum concentration of the lipid-free biomass was lower (4.3 ± 0.3 g L^–1^; Fig. 2b), and after 72 h the lipid-free biomass did not grow as glucose had been consumed.

As was observed in the medium M1 (Fig. 1), the high DO level (DO = 20%) promoted accumulation of total lipids in the biomass (Table S2; see Supplemental Material). Thus, the final concentration of total lipids (1.0 ± 0.1 g L^–1^) (Fig. 2a) was 2-fold greater than in the culture with the lower DO level (DO = 10%) (Fig. 2b). In all cultures in Fig. 2, the final concentration of the Or-N was the same (1.1 ± 0.1 g L^–1^). At the high DO level (DO = 20%), the residual Or-N allowed growth of the lipid-free biomass after glucose had been consumed (Fig. 2a).

DHA was the main fatty acid (30–50% of the total fatty acids) in the biomass grown using the medium M2 (Fig. 2c, d). The concentration of DHA was influenced by the DO level of the culture: the highest DHA concentration (145 ± 9 mg L^–1^) occurred near the end (145 h) of the culture if the DO level was 20% (Fig. 2c), but at the lower DO level (DO = 10%), the highest DHA concentration (67 ± 7 mg L^–1^) occurred at the end of the growth phase of the lipid-free biomass (Fig. 2d). The final concentration of EPA in the medium M2 with the DO set at 20% was 73 ± 5 mg L^–1^, whereas it was much lower (10 ± 2 mg L^–1^) at the DO setting of 10%.

**Fig. 3:**
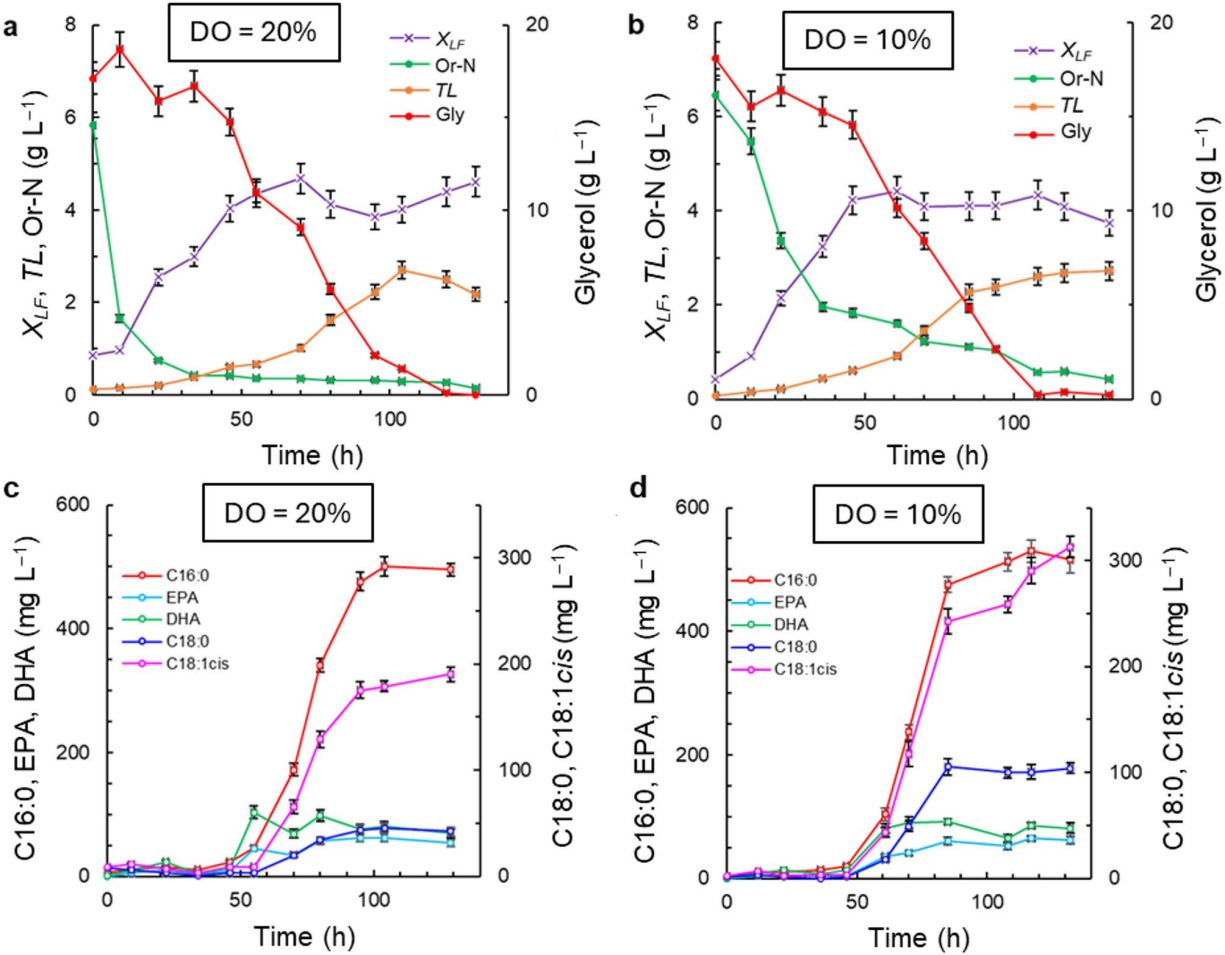
Effects of dissolved oxygen (DO) concentration on the time profiles of concentrations of the lipid-free biomass (*X*_*LF*_), glycerol (Gly), organic nitrogen (Or-N), total lipids (*TL*), and specific fatty acids (palmitic acid, C16:0; stearic acid, C18:0; oleic acid, C18:1cis; EPA; and DHA). The medium M3 was used with the following initial composition (g L^–1^): glycerol 20; yeast extract 6; and monosodium glutamate 0.6. The medium was made using a 1:1 v v^−1^ mixture of artificial seawater and distilled water

The RT2316-16 culture profiles in the medium M3 (Table 1) that used glycerol instead of glucose and DO levels of 10% and 20%, are shown in Fig. 3. With the high DO level (DO = 20%), the concentration of lipid-free biomass increased at an average rate of 0.079 g (L h)^–1^ during the first 46 h (Fig. 3a). The highest concentration of the lipid-free biomass was 4.7 ± 0.3 g L^–1^ at ~ 70 h (Fig. 3a). By the time glycerol had depleted to a low level (119 h), the concentration of the lipid-free biomass had declined to 3.9 ± 0.3 g L^–1^ (Fig. 3a). During rapid growth of the lipid-free biomass 87% of the original Or-N had been consumed by 22 h (Fig. 3a). Around 22 h, the total lipids in the biomass began to increase, reaching 40.3 ± 2.0% w w^–1^ in the dry biomass after 104 h (Table S3; see Supplemental Material). In this culture, the highest concentration of total lipids was 2.7 ± 0.2 g L^–1^. At the low DO level (DO = 10%) in the medium M3, the highest concentrations of the lipid-free biomass (4.4 ± 0.3 g L^–1^ at 61 h; Fig. 3b) and total lipids (2.7 ± 0.2 g L^–1^ at 132 h; Fig. 3b) were not significantly different (*p* > 0.05) than the concentrations in the high DO level culture (DO = 20%) (Fig. 3a).

In the high DO culture (DO = 20%), the fatty acid concentration did not exceed 65 mg L^–1^ during the first 64 h (Fig. 3c). During this period the total lipids content of the dry biomass was less than 17% w w^–1^ (Table S3; see Supplemental Material). After 64 h, the concentration of total fatty acids increased, reaching 1153 mg L^–1^ at 104 h, mainly due to an increase on concentration of C16:0 (17–44% of the total fatty acids) (Fig. 3c). The C16:0 concentration peaked after ~ 70 h once the growth of the lipid-free biomass had ceased (Fig. 3a, c). The average concentration of EPA between 55 h and 129 h was 52 mg L^–1^, whereas that of DHA during the same period was 83 mg L^–1^ (Fig. 3c). At the low DO level (DO = 10%), the patterns of changes in concentrations of fatty acids (Fig. 3d) were similar to those seen in the high-oxygen environment. In the low DO level culture (DO = 10%) stearic acid constituted ~ 17% of total fatty acids and oleic acid constituted ~ 31% (61–132 h) (Fig. 3d). In contrast, in the high DO level culture (DO = 20%) stearic acid amounted to ~ 5% of total fatty acids and oleic acid constituted ~ 20% of the total fatty acids (55–129 h) (Fig. 3c).

### Effect of the lupine extract as organic N source

**Fig. 4:**
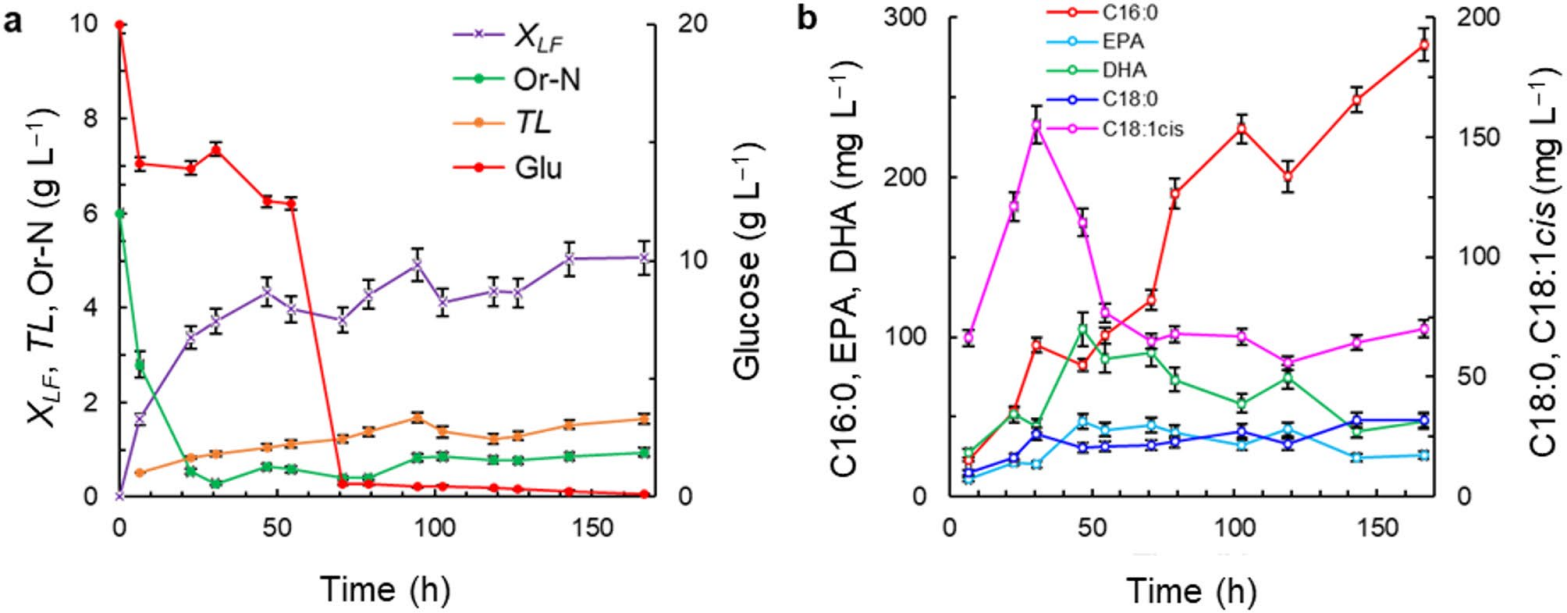
Time profile of the concentrations of lipid-free biomass (*X*_*LF*_), glucose (Glu), organic nitrogen (Or-N), total lipids (*TL*), and specific fatty acids (palmitic acid, C16:0; stearic acid, C18:0; oleic acid, C18:1cis; EPA; and DHA). The initial composition of the culture medium was as follows (g L^–1^): glucose 20 and lupine extract equivalent to Or-N concentration of 6 g L^−1^. The medium was made using a 1:1 v v^−1^ mixture of artificial seawater and distilled water. The DO concentration was 20% of air saturation

The hydrolyzed lupine extract was evaluated as a source of Or-N instead of the more expensive yeast extract and MSG (medium M4; Table 1). The DO level was controlled at 20% of air saturation. The resulting culture profiles are shown in Fig. 4.

The lupine extract Or-N was consumed rapidly: a > 50% consumption within the first 24 h (Fig. 4a). Afterwards, the average residual concentration of Or-N was 0.7 g L^–1^ (Fig. 4a). Glucose was consumed relatively slowly during 7–54 h, and after 71 h it had declined to around 0.6 g L^–1^ (Fig. 4a). The lipid-free biomass grew relatively rapidly until around 50 h (Fig. 4a); the average growth rate (0–47 h) of the lipid-free biomass was 0.089 g (L h)^–1^. The final concentration of the lipid-free biomass was 5.0 ± 0.9 g L^–1^ (Fig. 4a). This final concentration was 30% greater than the concentration obtained with the medium M1 and the same DO level (Fig. 1a). The concentration of total lipids continued to increase after cessation of growth of the lipid-free biomass (Fig. 4a). The final concentration of total lipids was 1.7 ± 0.1 g L^–1^ (Fig. 4a). The main fatty acid in the biomass was C16:0 (Fig. 4b). The final concentration of C16:0 was 280 ± 12 mg L^–1^ at 167 h (Fig. 4b). The concentrations of C18:1, EPA and DHA increased until 33 h, the time at which the growth of the lipid-free biomass ceased (Fig. 4a). The concentrations of C18:1 and DHA declined once glucose was exhausted and while the C16:0 concentration still increased.

### Fed-batch culture

**Fig. 5:**
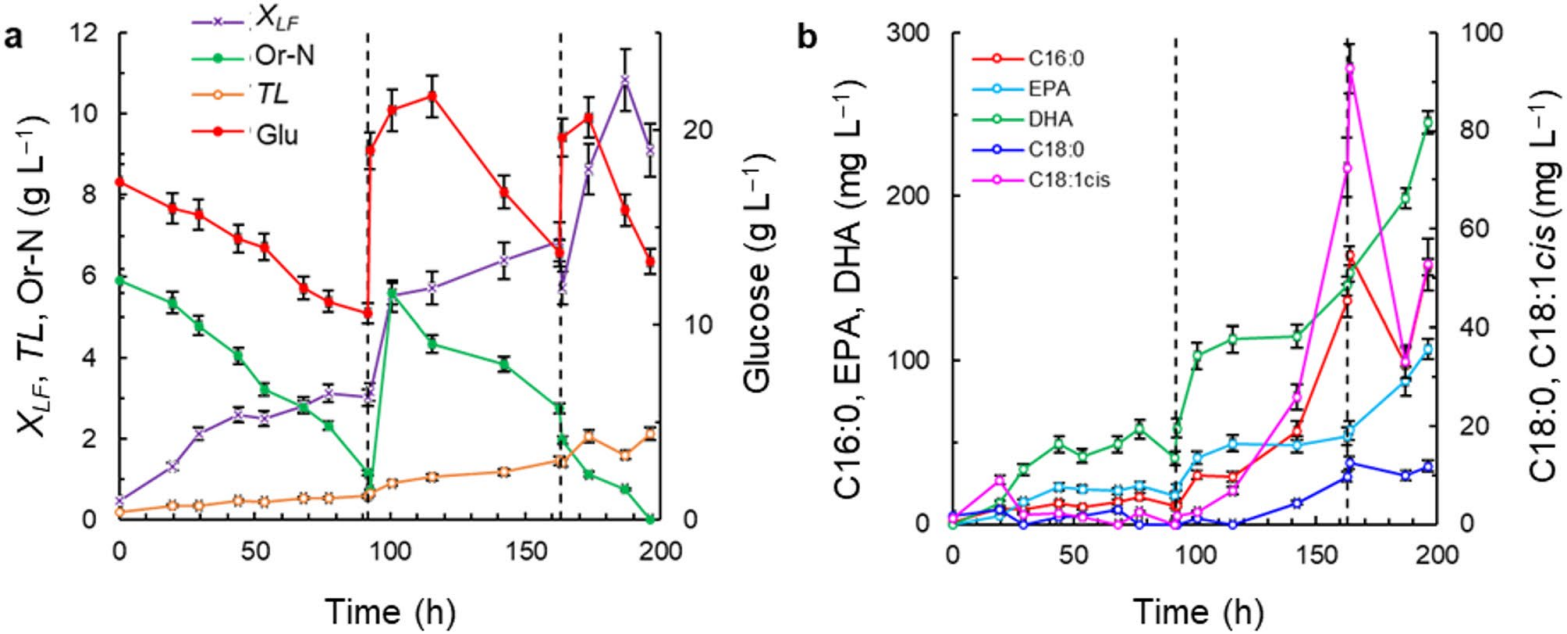
Time profiles of concentrations of the lipid free biomass (*X*_*LF*_), glucose (Glu), organic nitrogen (Or-N), total lipids (*TL*), and specific fatty acids (palmitic acid, C16:0; stearic acid, C18:0; oleic acid, C18:1cis; EPA; and DHA) in fed-batch culture. The initial composition of the medium M1 was the following (g L^–1^): glucose 20; yeast extract 6; and monosodium glutamate 0.6. The medium was made using a 1:1 v v^−1^ mixture of artificial seawater and distilled water. After 92 h, the culture was fed with a concentrated solution (250 mL) containing glucose, yeast extract and monosodium glutamate. After 163 h, the culture was fed with a concentrated solution (250 mL) containing only glucose. The vertical dashed lines demarcate the feeding times. The DO concentration was 20% of air saturation

In the fed-batch culture with the DO level controlled at 20% of air saturation, the preceding batch stage initiated with the M1 medium, the first feeding (250 mL of concentrated glucose and Or-N (yeast extract and MSG)) occurred at 92 h and the second feeding (250 mL, glucose only) occurred at 163 h. The data are shown in Fig. 5.

In the batch phase (i.e., before the first feeding), the growth was slow (Fig. 5a) as had been observed earlier in batch cultures with the medium M1 in combination with a DO level of 20% (Fig. 1a). The first feeding increased the concentrations of the nutrients (Or-N and glucose), resulting in resumed growth from a near lag phase just prior to the feeding (Fig. 5a). In the period between the two feedings (i.e., 92–163 h; Fig. 5a) the concentration of the lipid-free biomass increased nearly 2.3-fold from 3.0 ± 0.2 g L^–1^. The second feeding resulted in a further rapid increase in the concentration of the lipid-free biomass, notwithstanding the initial drop in concentration (at 163 h) due to the dilution associated with the feeding (Fig. 5a). The peak concentration of the lipid-free biomass (10.8 ± 0.6 g L^–1^) was reached at 187 h (Fig. 5a). The concentration of the total lipids increased from 0.2 ± 0.1 g L^–1^ to 0.5 ± 0.1 g L^–1^ near the end of the initial batch phase, and then further to 1.5 ± 0.1 g L^–1^ near the end of the first fed-batch phase, and to 2.1 ± 0.2 g L^–1^ at the end of the second fed-batch phase (Fig. 5a).

The main fatty acid in the biomass was DHA (Fig. 5b). The concentration of DHA increased sharply after the second feeding to ultimately reach 245 ± 11 mg L^–1^ (Fig. 5b). The time profile of the concentration of EPA was similar to that of DHA, and the final concentration of EPA was 107 ± 8 mg L^–1^ (Fig. 5b). The other fatty acids (C16:0, C18:0, C18:1) also increased in concentration after the first feeding (Fig. 5b).

Summarizing, the effect of DO level on growth and lipid production depended on medium composition (Table 2). A high DO level (20%) promoted the growth of the lipid-free biomass in medium M2 (high concentration of Or-N) and medium M4 (lupine extract) whereas in medium M1 (high concentration of glucose) it promoted lipid accumulation due to arrested growth. The DO level had a lesser effect on growth and lipid production when glycerol was the carbon source (medium M3). Media M1 and M2 in combination with high DO levels favored the synthesis of EPA and DHA. The fed-batch operation was found to be suitable for enhancing the concentrations of EPA and DHA.

**Table 2:** Summary results of batch and fed-batch cultures. The values in parentheses are the times (h) at which the concentrations were measured

Medium	DO (%)	X_LF_ (g L^−1^)	TL (g L^−1^)	EPA (mg L^−1^)	DHA (mg L^−1^)	SQ (mg L^−1^)
M1	5	5.8 ± 0.4 (94)	1.2 ± 0.5 (70)	43 ± 4 (129)	88 ± 9 (57)	786 (94)
M1	20	3.9 ± 0.2 (149)	2.9 ± 0.2 (149)	224 ± 12 (30)	550 ± 25 (44)	1485 (149)
M2	10	4.3 ± 0.3 (72)	0.7 ± 0.1 (72)	10 ± 2 (72)	67 ± 7 (72)	157 (24)
M2	20	5.9 ± 0.4 (120)	1.0 ± 0.1 (120)	73 ± 5 (111)	145 ± 9 (145)	96 (96)
M3	10	4.4 ± 0.3 (61)	2.7 ± 0.2 (132)	65 ± 4 (117)	91 ± 5 (85)	329 (46)
M3	20	4.7 ± 0.3 (70)	2.7 ± 0.2 (104)	62 ± 6 (95)	103 ± 10 (55)	831 (80)
M4	20	5.0 ± 0.9 (167)	1.7 ± 0.1 (167)	47 ± 5 (47)	105 ± 10 (47)	202 (167)
Fed-batch	20	10.8 ± 0.6 (187)	2.1 ± 0.2 (173)	107 ± 8 (196)	245 ± 11 (196)	332 (101)

Abbreviations: DO, dissolved oxygen; *X*_LF_, lipid-free biomass; *TL*, total lipids; EPA, eicosapentaenoic acid; DHA, docosahexaenoic acid; SQ, squalene

### Effects of dissolved oxygen concentration and the composition of the growth medium on squalene in the biomass

**Fig. 6:**
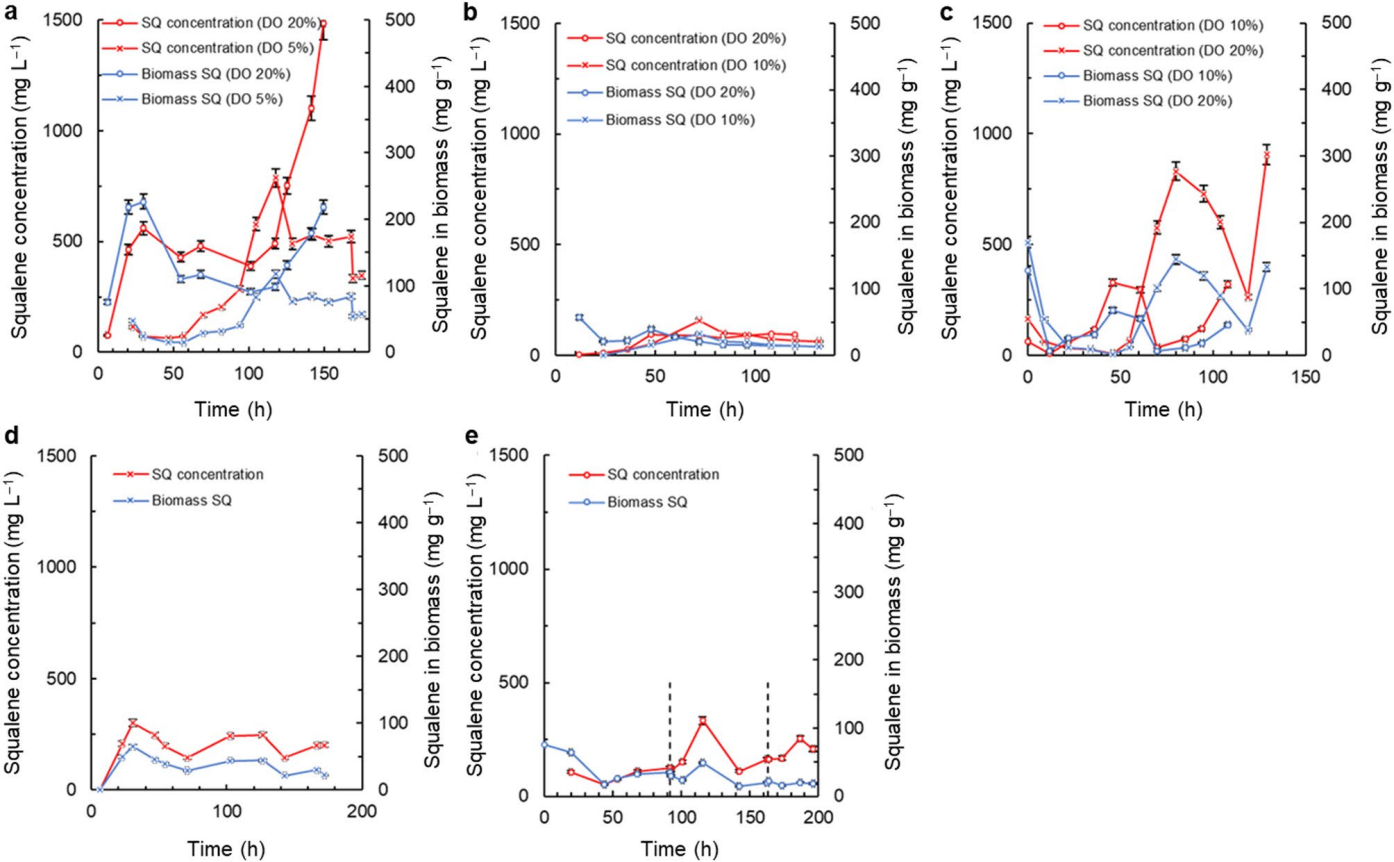
Time profiles of squalene (SQ) concentration and squalene content in the biomass during cultures in different growth media at different dissolved oxygen (DO) concentrations: (**a**) medium M1 with DO concentrations of 5% and 20%; (**b**) medium M2 with DO concentrations of 10% and 20%; (**c**) medium M3 with glycerol as the carbon source and DO concentrations of 10% and 20%; (**d**) medium M4 with lupine extract, and DO concentration of 20%; and (**e**) fed-batch culture with DO concentration of 20%. The vertical dashed lines in (**e**) demarcate the feeding times

The squalene content in the biomass and the squalene concentration in the culture broth were determined for the various culture conditions discussed in the earlier sections (Figs. 1, 2, 3, 4 and 5). The data are shown in Fig. 6. The high DO level (DO = 20%) positively affected the squalene content of the biomass grown in the medium M1 (Fig. 6a). During the first growth phase of the lipid-free biomass (Fig. 1a), the squalene content in the biomass increased but once the lag phase commenced, there was a decline in the squalene content of the biomass (Fig. 6a). When the lipid-free biomass growth resumed at a relatively slower pace compared to earlier (Fig. 1a), its squalene content increased (Fig. 6a). At the end of the culture, the squalene content of the biomass peaked at 218 ± 11 mg g^–1^ (Fig. 6a). In this same culture, the final concentration of squalene was 1.49 ± 0.06 g L^–1^ (Fig. 6a). In the medium M1, with the low DO level (DO = 5%), the squalene content of the biomass increased after 57 h paralleling the growth of the lipid-free biomass and finally reached 118 ± 6 mg g^–1^ once the lipid-free biomass had ceased to grow (Fig. 1b). In this culture, the average squalene concentration towards the end was 454 ± 90 mg L^–1^ (Fig. 6a).

The biomass grown in the medium M2 had a low content of squalene (14–57 mg g^–1^) (Fig. 6b). After 120 h, the squalene concentration was 95 ± 8 mg L^–1^ under the high DO (DO = 20%) condition, but it was much lower (67 ± 4 mg L^–1^) under the low DO condition (DO = 10%) (Fig. 6b).

The DO level significantly affected (*p* < 0.05) the squalene content of the biomass grown using glycerol (Fig. 6c). Initially, the squalene content of the biomass decreased: with the low DO level (DO = 10%) the squalene content declined from 128 ± 6 mg g^–1^ to 7.2 ± 1 mg g^–1^ whereas a similar decline took place over a longer period (the first 34 h) with the high DO level (DO = 20%). Subsequently, the squalene content rose to 68 ± 4 mg g^–1^ and 145 ± 7 mg g^–1^ in the low and high DO conditions, respectively. The highest squalene concentration (0.91 ± 0.05 g L^–1^ at 129 h) occurred in the culture with the high DO level (DO = 20%) (Fig. 6c). This concentration was 2.8-fold greater than the maximum concentration in the low DO level (DO = 10%) culture (Fig. 6c).

The biomass grown using the lupine extract had a relatively low squalene content (an average of 39 ± 13 mg g^–1^) and the average final squalene concentration in this culture was 213 ± 48 mg L^–1^ (Fig. 6d). In the fed-batch culture, the average squalene content of the biomass (Fig. 6e) was 41 ± 23 mg g^–1^ at the end of the batch phase, 27 ± 12 mg g^–1^ after the first feeding, and 18 ± 2 mg g^–1^ after the second feeding. Average squalene concentration in this culture was 185 ± 71 mg L^–1^ (Fig. 6e).

## Discussion

Some thraustochytrids naturally produce EPA, DHA, and the triterpenoid hydrocarbon squalene. Therefore, these microorganisms can be used for producing these compounds after suitable strains and culture conditions have been identified. Some of these products are currently sourced from oils of wild-caught marine fish, a mode of production that is considered ecologically unsustainable (Chi et al. 2022). For example, most of the squalene is obtained from shark liver oil (Patel et al. 2022). Potentially sustainable alternative sources are microbial oils such as thraustochytrid lipids. Commercial production of thraustochytrid-derived DHA has proven successful (Chi et al. 2022), suggesting that other microbial oils have the potential to be commercialized. Hence the rationale for the present study on production of squalene and omega-3 long chain polyunsaturated fatty acids by the novel thraustochytrid RT2316-16 using different combinations of media and DO concentrations. Although genetically modified microorganisms have been developed for products such as squalene (Patel et al. 2022), EPA (Amiri-Jami et al. 2014; Cao et al. 2012) and DHA (Amiri-Jami et al. 2014), their commercial use in food and feed applications is either highly regulated (Hanlon and Sewalt 2020; Wesseler et al. 2023) or not permitted in some jurisdictions.

### Production of biomass and fatty acids by RT2316-16

The quantity and composition of the lipids in microbial biomass is highly sensitive to the nutrients provided in the culture medium and the other culture conditions. In *Thraustochytrium* sp. RT2316-16, the focus of the present study, the culture conditions were previously shown to affect the production of EPA, DHA, carotenoids, and phospholipids in shake flasks (Leyton et al. 2022; Valdebenito et al. 2022). Shake flask culture does not typically allow any level of control of the concentration of dissolved oxygen (DO) in the medium. Therefore, the present work was carried out in a stirred tank bioreactor at various controlled concentrations of DO to elucidate the impact of oxygen concentration on the production of total lipids, fatty acids and squalene. As the concentration of metabolically active microbial biomass increased with growth, the volumetric oxygen consumption rate in the culture increased. To compensate for the increased consumption of oxygen, the oxygen transfer rate in the bioreactor was automatically increased to ensure that the measured dissolved oxygen concentration remained at the controlled value. A cascaded control combining increased agitation speed of the mixing impeller and the air flow rate was used to achieve effective control of the dissolved oxygen concentration.

The growth kinetics of the lipid-free biomass and the production of total lipids were found to be strongly influenced both by the DO concentration and the nutritional composition of the culture medium. For growth in the medium M1, a relatively high DO level (DO = 20% of air saturation) promoted accumulation of total lipids in the biomass (Table S1; see Supplemental Material) compared to low DO level (DO = 5%). Although the low DO level (DO = 5%) satisfactorily supported the growth of the lipid-free biomass, the total lipids in the biomass were reduced.

In oleaginous microorganisms, the accumulation of triacylglycerol lipids typically occurs after cell growth has ceased due to exhaustion of some key nutrient (e.g., nitrogen, phosphorus) other than the carbon source. Once a key non-carbon nutrient is missing, the available organic carbon can no longer support cell growth but continues to be used to build up reserves of lipids that do not contain the missing nutrient. For example, triacylglycerols are comprised of only carbon, oxygen and hydrogen. Similarly, squalene is comprised of only carbon and hydrogen. The cessation of growth of the lipid-free biomass in the medium M1 with the DO controlled at 20% was not due to a depletion of glucose or organic nitrogen (Or-N) (Fig. 1a). The observed cessation of growth may have been due to possible production of some growth-inhibitory metabolite, or generation of reactive oxygen species in a high-oxygen environment, and/or the depletion of some essential component in the Or-N fraction of the nitrogen source (yeast extract). The arrest of the lipid-free biomass growth was transient, and the growth resumed after a short period (Fig. 1a). A transient arrest of growth, or a diauxic growth (or diauxie) pattern, is consistent with complete consumption of a readily metabolizable nutrient such as a component of Or-N, followed by an adaptation period of little or no growth during which the microorganism builds the enzyme machinery necessary for metabolizing the other available nitrogen-containing components within the Or-N mixture.

Compared to the medium M1, the medium M2 had a higher concentration of Or-N but less glucose. In M2, the effect of the DO concentration on production of the lipid-free biomass was opposite to that seen in the medium M1, i.e. the high DO level (DO = 20%) promoted the growth of lipid-free biomass and the phenomenon of transient arrested growth did not occur. This concurred with the earlier inference that depletion of some component of Or-N was responsible for the temporarily arrested growth in the medium M1 that was low in Or-N compared to the medium M2.

Regardless of the DO level and the initial concentrations of glucose and Or-N, the rate of consumption of Or-N showed sharp variations at certain times during the batch culture. This was likely related to preferential consumption of some components of the yeast extract, with the others being consumed after the preferred component was no longer available. These changes in the nitrogen components being metabolized may have promoted an increased accumulation of total lipids in the biomass and may also have affected the composition of the fatty acids in the lipids (Figs. 1 and 2).

In earlier studies in shake flasks without control of the DO concentration, the concentration of the lipid-free biomass in medium M1 was 4.3 g L^–1^ and the concentration of the total lipids was 1.7 g L^–1^ (Valdebenito et al. 2022). Also in shake flasks, the concentration of the lipid-free biomass in medium M2 was 3.7 g L^–1^ and the concentration of the total lipids was 0.4 g L^–1^ (Valdebenito et al. 2022). These concentrations were comparable to those obtained in the present work with a low DO level (DO = 5%) in medium M1 (i.e., lipid-free biomass concentration = 4.9 g L^–1^, and total lipids = 1.2 g L^–1^). In the less oxygenated environment (DO = 10%), in medium M2 in the present work, the lipid-free biomass concentration was 4.3 g L^–1^, and the total lipids concentration was and 0.5 g L^–1^. This comparison suggests that an accurate evaluation of the potential of thraustochytrids as producers of lipids requires controlled oxygen cultures; the conventional shake flask culture is unsatisfactory. As thraustochytrids are obligate aerobes, the oxygen concentration in a culture can reasonably be expected to critically influence their metabolism, as observed in the present work.

The data showed that the concentrations of EPA and DHA in cultures with controlled DO followed similar time-dependent variations. This may suggest that DHA was produced via elongation–desaturation pathway in which EPA is an intermediary; however, certain omega-3 fatty acids (e.g., a-linoleic acid, C18:3^D9,12,15^; stearidonic acid, C18:4 ^D6,9,12,15^; and eicosatetraenoic acid, C20:4 ^D8,11,14,17^) of the elongation–desaturation pathway were not found in RT2316-16. Therefore, in RT2316-16 EPA and DHA were most likely synthesized from the omega-6 fatty acid arachidonic acid (C20:4 ^D5,8,11,14^) as has been reported in some unrelated photosynthetic marine microorganisms (Guihéneuf et al. 2013).

Arachidonic acid was one of the PUFAs produced by RT2316-16 at low levels (not shown). In the elongation–desaturation pathway fatty acid desaturases introduce double bonds in saturated, monounsaturated and polyunsaturated fatty acids. In the synthesis of DHA and EPA from C18:0, three desaturases are involved: the delta-9 desaturase, that acts on a saturated fatty acid, and the delta-5 (D5D) and delta-6 desaturases (D6D) that act on specific PUFA. All fatty acid desaturases require molecular oxygen and a reducing agent (NAD(P)H and NADH). Fatty acid elongation utilizes malonyl-CoA, as a two-carbon donor, and NADPH as a reducing agent (Cook and McMaster 2002). This oxygen-dependence probably explains the relatively high concentrations of EPA and DHA observed in the cultures with the high DO level (DO = 20%; Figs. 1c and 2c), and the low concentration of the PUFA in medium M2 in which the low concentration of glucose (Table 1) may have reduced the availability of the malonyl-CoA required for fatty acid elongation. In the medium with glycerol, irrespective of the DO level, concentration of all fatty acids remained below 70 mg L^−1^ (Fig. 3c, d) during the growth phase of the lipid-free biomass (Fig. 3a, b), suggesting that the carbon source and the co-factors were channeled to biomass growth. However, as soon as the biomass growth ceased, fatty acids (C16:0, C18:0 and C18:1) were synthesized. At this point fatty acid desaturation and elongation were likely constrained by the availability of the co-factors (NADH and NAD(P)H) and malonyl-CoA as glycerol had been consumed (Fig. 3a, b). The initial trends in the concentrations of EPA and DHA in Fig. 5b suggest that maintaining a high concentration of the substrates in the medium (Fig. 5a) might be a possible strategy for enhancing the production of these fatty acids. Furthermore, the glycerol-based medium M3 contained 50% less elemental carbon in the main carbon source than the glucose-based medium M1 with a glucose (C_6_H_12_O_6_) concentration that was identical to the glycerol (C_3_H_8_O_3_) concentration in medium M3.

The lupine extract proved to be a good source of Or-N for the growth of RT2316-16: it supported a higher growth rate than was obtained with the combination of yeast extract and MSG (Table 2), and a higher final concentration of the lipid-free biomass. Although the concentration of the lupine Or-N decreased rapidly (< 50 h), it was not fully exhausted possibly because the residual fraction required the presence of glucose that had been almost fully consumed by this point. Generally good results were observed with lupine extract as a sole nitrogen source because lupine flour is known to contain all the same amino acids (Tomczak et al. 2018; Wasilewko and Buraczewska 1999) as found in yeast extract (Tao et al. 2023). In addition, lupine flour is known to provide many of the nitrogenous vitamins (e.g., vitamins B1, B2, B5, B6, and B9) (Siitonen et al. 2024) found in yeast extract (Tao et al. 2023).

Lupine extract was a cheaper source of Or-N compared to yeast extract. For example, the media M1 and M3 that had the lowest quantities of yeast extract and MSG may be compared with the medium M4. Considering only the cost of the nitrogen sources, both M1 and M3 would cost around US$ 679 m^−3^ based on a wholesale yeast extract (food-grade) price of around US$ 112 kg^−1^ and a wholesale MSG (food-grade) price of US$ 12 kg^−1^. In contrast, the estimated cost of the medium M4 was US$ 95.18 m^−3^ based on the following: a 50 kg requirement of lupine flour at a market price of US$ 1.9 kg^−1^ and a protease enzyme requirement of 35 L equating to a total enzyme activity of around 595,000 U. Food-grade microbial (*Bacillus subtilis*) protease enzymes needed for producing the lupine flour hydrolysate can be purchased typically for US$ 30 kg^−1^ with an enzyme activity of 10^5^ U g^−1^. Thus, the cost of the required 595,000 U of enzyme activity would be no more than US$ 0.18. Based on this analysis of costs, the cost of medium M4 would be around 14% of the cost of either the medium M1, or the medium M3. The medium M2 with twice as much yeast extract and MSG as the medium M1 or M3, would cost around US$ 1358 m^−3^.

The feeding regimens used in the fed-batch fermentations were designed to initially (first feeding) rapidly increase the concentration of the lipid-free biomass and subsequently to slow growth and promote lipid synthesis during the second feeding. The arrested growth seen in medium M1 (Fig. 1a, and Fig. 5a before the feeding) was reversed when the concentration of Or-N increased resulting in simultaneous production of EPA and DHA. The significant increases in concentrations of EPA and DHA after the second feeding were ascribed to the availability of resources for desaturation and elongation reactions: a high DO level (DO = 20%) and actively growing cell in which glycolysis could produce the reducing agent and the pyruvate needed for the synthesis of citrate in the tricarboxylic acid cycle. The citrate could then be used in the cytosol to produce acetyl-CoA and malonyl-CoA. The DHA concentration in the fed-batch culture was lower than in the batch culture (Fig. 1c), suggesting that the feed composition (ratio of carbon-to-nitrogen) and the feeding frequency, or rate, needed optimization.

Often, the volumetric productivity of a substance (biomass, or product) is used for comparing the performance of different fermentations. Volumetric productivity of a substance in batch and fed-batch fermentations is calculated as the maximum concentration of the substance divided by the time required to attain it. The data shown in Table 2 could be used to calculate the productivities of the biomass and the other metabolites (total lipids, EPA, DHA, squalene) for each of the eight fermentation scenarios summarized in Table 2. Based on the calculated productivities (not shown), the fed-batch operation was always inferior to the best batch operation for attaining the maximum productivity of a target substance. The batch operations providing the highest volumetric productivities were the following: (1) the medium M3 with 10% DO for the biomass (productivity = 0.072 g L^−1^ h^−1^); the medium M3 with 20% DO for total lipids (productivity = 0.026 g L^−1^ h^−1^); the medium M1 with 20% DO for EPA (productivity = 7.467 mg L^−1^ h^−1^); and the medium M1 with 20% DO for DHA (productivity = 12.500 mg L^−1^ h^−1^). As noted earlier, the highest observed productivities of EPA and DHA in cultures that had high concentrations of DO (DO = 20%) were likely indicative of the oxygen-dependent elongation–desaturation pathway contributing to their synthesis. A batch culture proved to be best for maximizing the productivity of squalene as discussed in the next section.

### Production of squalene by RT2316-16

Squalene is an intermediate metabolite in the sterol synthesis pathway. The products of this pathway influence properties of cell membranes such as fluidity and permeability. Thraustochytrids are known to produce sterols (Bi et al. 2023; Ishibashi et al. 2023; Menzorov et al. 2024), although there is no specific information on production of sterols by *Thraustochytrium* sp. RT2316-16. In related thraustochytrids such as *Schizochytrium* (now *Aurantiochytrium*; Chi et al. 2022) sp. S31, sterols such as cholesterol, stigmasterol, lanosterol, and cycloartenol are produced (Bi et al. 2023). The biosynthesis of sterols and fatty acids, particularly DHA, in some thraustochytrids appears to be co-regulated. For example, genetic modifications for increased production of DHA have reduced production of squalene and sterols in *Schizochytrium* (now *Aurantiochytrium*; Chi et al. 2022) sp. HX-308 (Ren et al. 2015). Conversely, modifications to the mevalonate pathway for increased production of squalene and carotenoids have reduced the amount of DHA in the biomass of *Schizochytrium* sp. HX-308 (Huang et al. 2021). Whether squalene accumulates in the biomass, decreases, or remains unchanged in concentration depends on relative rates of its production and consumption under the prevailing culture scenarios.

In the absence of nutrient limitations (including oxygen limitation), squalene is unlikely to accumulate in the biomass as it does not participate in other metabolic pathways. Nonetheless, an imbalance between the rate of synthesis of squalene and its oxidation by squalene monooxygenase or squalene epoxidase (Bi et al. 2023) could result in accumulation of squalene. The rates of enzymatic reactions depend both on the concentrations of the enzymes and the concentrations of the substrates, farnesyl pyrophosphate and squalene in this case. Farnesyl pyrophosphate is produced in the mevalonate pathway in which 3-hydroxy-3-methylglutaryl-coenzyme A reductase (HMGR) is the rate limiting enzyme in *Aurantiochytrium* sp. 18 W-13a (Yang et al. 2022). The HMGR activity in the strain 18 W-13a strongly correlated with the squalene content. If a similar scenario applied to RT2316-16, anything that increased the flux through the mevalonate pathway (e.g., an excess of acetyl-CoA, the starting metabolite of the pathway), would promote accumulation of squalene.

Availability excess acetyl-CoA is also necessary for accumulation of fatty acids, as was consistent with the observations in media M1, M2 and M3. The low concentration of glucose in medium M2 (Table 1) did not support sufficient production of acetyl-CoA and thus the lipid content of the biomass was low and fatty acids and squalene did not accumulate. Similarly, in medium M4 that promoted a rapid growth of the biomass, glucose was fully consumed and therefore there was no subsequent synthesis of squalene. In contrast, the media M1 and M3 with high concentrations of either glucose or glycerol (Table 1), promoted accumulation of total lipids, fatty acids and squalene when the DO concentration was high (DO = 20%; Fig. 6a, c). The squalene concentrations in these two cases (Table 2) were comparable to values reported in cultures of the following other thraustochytrids (Table 3): *Aurantiochytrium* sp. 18 W-13a (Kaya et al. 2011), *Schizochytrium* sp. S31 (Schütte et al. 2024), and *Thraustochytrium* sp. Yonez5-1 (Nakazawa et al. 2014). Note that total lipids (including EPA, DHA and squalene) are intracellular components of the biomass and their relatively low concentrations (Table 2) were a direct consequence of the low concentration of the biomass of RT2316-16 (Table 2) compared to the concentrations that have been reported for other thraustochytrids in fed-batch cultures.

In earlier work, a genetically modified *Schizochytrium* sp. HX-308 was shown to provide a squalene titer of 13.73 g L^−1^ (Table 3) in a fed-batch that had a dry biomass concentration of 96 g L^−1^ (Xu et al. 2025), equivalent to a biomass specific squalene production level of 0.14 g g^−1^. Compared with this, the squalene content of the dry biomass of RT2316-16 grown in the medium M1 under a high DO level (DO = 20%) was 0.22 ± 0.01 g g^−1^ (Fig. 6a). Thus, the wildtype RT2316-16 used in the present work was ~ 57% richer in squalene than the *Schizochytrium* sp. that had been engineered to overproduce it. Indeed, in terms of the squalene content of the biomass, RT2316-16 grown as specified earlier in this paragraph, was better than the other thraustochytrids for which the specific production data are shown in Table 3. Based on the data in Table 2, the highest productivity (10.39 mg L^−1^ h^−1^) of squalene was attained in batch culture using the medium M3 with a DO level of 20%.

**Table 3:** Production of squalene by some thraustochytrids

Thraustochytrid	C and *N* sources	Squalene concentration	Reference
*Aurantiochytrium* sp. BR-MP4-A	G/YE/MSG	0.57 mg g^−1^ (biomass)	Li et al. 2009
*Aurantiochytrium* sp. 18 W-13a	G/YE/P	1.29 g L^−1^	Kaya et al. 2011
*Aurantiochytrium* sp. TWZ-97	G/YE/MSG	0.19 g L^−1^	Zhang et al. 2019
*Aurantiochytrium* sp. T66	^a^AFW_C_	0.93 g L^−1*^	Patel et al. 2020a
*Aurantiochytrium* sp. 18 W-13a^GM^	G/YE/T	10.8 mg g^−1^ (biomass)	Yang et al. 2022
*Aurantiochytrium* sp. TWZ-97	^h^G/YE/MSG	0.24 g L^−1^	Ali et al. 2023
*Aurantiochytrium* sp.	G/YE/P/AM	0.055 g L^−1^	Furlan et al. 2025
*Schizochytrium* sp. PQ6	G/YE	98.07 mg g^−1^ total lipid^FB^	Hoang et al. 2018
*Schizochytrium limacinum* SR21	^b^AFW_C_	69.30 mg g^−1^ biomass	Patel et al. 2020b
*Schizochytrium* sp. SR21	^e^G/NS/MSG	0.007 g L^−1^	Duan et al. 2023
*Schizochytrium* sp. HX-308	G/YE/MSG/AM	2.2 g L^−1^	Nong et al. 2024
*Schizochytrium* sp. S31	G/YE/P	1.13 g L^−1**^	Schütte et al. 2024
*Schizochytrium limacinum* SR21	G/YE	0.96 g L^−1**^	Shafiq et al. 2024
*Schizochytrium* sp. HX-308^GM^	^i^G	13.73 g L^−1**^	Xu et al. 2025
*Thraustochytrium* sp. Yonez5-1	G/T/YE	1.10 g L^−1^	Nakazawa et al. 2014
*Thraustochytrium* ATTC 26,185	G/YE/MSG	0.124 g L^−1^	Zhang et al. 2021
*Thraustochytrium* ATCC 26,185	^c^G/YE/MSG	0.62 g L^−1^	Ali et al. 2022
*Thraustochytrium* ATCC 26,185	G/YE	0.46 g L^−1^	Zhang et al. 2022
*Thraustochytrium* sp.	G/YE/P/AM	0.026 g L^−1^	Furlan et al. 2025
*Thraustochytium* sp. RT2316-16	G/YE	1.49 ± 0.06 g L^−1^	This work

Abbreviations: G, glucose; YE, yeast extract; MSG, monosodium glutamate; P, peptone; T, tryptone; AM, ammonium sulfate; NS, sodium sulfate; AFW_C_, agri-food carbon source; AFW_N_, agri-food waste nitrogen source

Superscripts: *, AFW medium was better than laboratory medium; **, AFW medium was worse than laboratory medium; FB, fed-batch mode; a, food leftover; b, hydrolyzed forest biomass; c, mannitol-biotin supplementation; d, black liquor; e, corn steep powder and glycerol; f, orange peels; g, hydrolyzed thraustochytrid biomass; h, alpha tocopherol; i, sesamol; GM, genetically modified

The effect of the DO concentration on the squalene content in the biomass of RT2316-16 was different compared to data reported for other thraustochytrids. For example, in the thraustochytrid ACEM 6063, possibly *Schizochytrium* sp. (or *Aurantiochytrium* sp.), a low level of DO (DO = 5%) promoted squalene accumulation to around 2 mg g^–1^ in the biomass (Lewis et al. 2001). Squalene accumulation under low oxygen was hypothesized to be associated with oxygen inhibition of certain downstream enzymes that consumed squalene to generate the other metabolites needed for the synthesis of sterols (Lewis et al. 2001).

The fed-batch culture (Fig. 5) with the tested feeding strategies did not prove useful for producing squalene-rich biomass, possibly because there was no nutrient limitation as was evidenced by a continuous increase in the concentration of the lipid-free biomass during the entire culture. The low lipid content of the biomass further suggested a low availability of acetyl-CoA for the synthesis of fatty acids and also for the synthesis of squalene. In the latter case, the rate of squalene synthesis was likely comparable to the rate of its consumption by the action of SQE for use in production of sterols that was needed to form the cell membranes of the growing biomass.

In studies with a different thraustochytrid (*Schizochytrium* sp. S31; Schütte et al. 2024) squalene production was compared among batch, fed-batch and continuous culture operations. Continuous culture implemented with a relatively high dilution rate (dilution rate = 0.025 h^− 1^) resulted in biomass with the highest squalene content (39.7 ± 1.3 mg g^–1^). In a steady-state continuous culture, the specific growth rate of the biomass is identical to the dilution rate (Chisti 2010), meaning that the growth rate was high. In such high dilution rate cultures, the steady state concentrations of all nutrients in the culture vessel tend to be high, implying an absence of nutritional stress. Although the most squalene-rich biomass was produced in continuous culture, the highest concentration of squalene was produced in a fed-batch operation with pulsed feeding of glucose (Schütte et al. 2024). This suggests that a more comprehensive future evaluation of feeding strategies in fed-batch operations may be worthwhile for enhancing the productivity of squalene in *Thraustochytrium* sp. RT2316-16.

### Conclusions

The composition of the culture medium and the concentration of dissolved oxygen (DO) affected both the squalene content of the biomass and the composition of the fatty acids in the total lipids. A medium relatively rich in glucose compared to Or-N (the medium M1) in combination with a DO level of 20%, limited the growth of lipid-free biomass and favored the accumulation of both total lipids and squalene in the biomass. Replacing glucose with an equal mass concentration of glycerol in the medium M1 enhanced squalene accumulation in the biomass.

The specific nitrogen compounds present in the organic nitrogen source affected the composition of the lipids produced. The microorganism consumed lupine extract Or-N more rapidly compared to yeast extract, promoting the synthesis of palmitic acid and oleic acid to the detriment of EPA and DHA. Relatively high concentrations of the long-chain omega-3 fatty acids EPA and DHA could be obtained in oxygen-rich medium when glucose concentration was kept above a minimum threshold, for example, by using a fed-batch operation. In a 149-h batch operation, the maximum content of squalene in the biomass (218 mg g^−1^) occurred under the following conditions: a DO concentration of 20%, 15 °C, and the medium M1. These findings demonstrate that the metabolism of *Thraustochytrium* sp. RT2316-16 can be directed to the production of omega-3 PUFA, saturated fatty acids, or isoprenoids through the control of DO and the composition of the culture medium. The yields of lipids may be further improved in the future through optimization of feeding and/or engineering of the strain. Lupine extract proved to be a cheaper nitrogen source for promoting excellent biomass growth, but not for maximizing the contents of specific lipids in the biomass. Nonetheless, this would be significant in applications (e.g., use of thraustochytrid biomass in aquaculture feeds) where the whole biomass is wanted, rather than individual metabolites. Depending on the culture conditions used, *Thraustochytrium* sp. RT2316-16 has the potential to be developed as nutritionally rich wholesome biomass for use as aquaculture feed, as a producer of microbial oils (total lipids), or as a producer of specific lipids (DHA, EPA, squalene).

## Supplementary Information

Below is the link to the electronic supplementary material.

Supplementary Material 1

Supplementary Material 2

## Data Availability

The data supporting the findings of this study are available from the corresponding author upon reasonable request.
