# Supplementary material for: Production of squalene and fatty acids by Thraustochytrium sp. RT2316-16: effects of dissolved oxygen and the medium composition
Source: Bioresour Bioprocess. 2025 Sep 16;12(1):98. doi: 10.1186/s40643-025-00937-x (PMC12436261; doi:10.1186/s40643-025-00937-x)
Supplement: Supplementary file 1 — Supplementary Material 1 [file 40643_2025_937_MOESM1_ESM.docx]

**Supplemental Material**

**Production of squalene and fatty acids by *Thraustochytrium* sp. RT2316-16: Effects of dissolved oxygen and the medium composition**

Paris Paredes, Liset Flores, Mariela Bustamante, Yusuf Chisti, Juan A. Asenjo, Carolina Shene

*S1. Composition of the chemically defined media used for growth*

The minerals salts solution comprised of the following (g L^−1^ deionized water): 0.3 MnCl_2_·4H_2_O, 0.3 ZnSO_4_·7H_2_O, 0.004 CoCl_2_·6H_2_O, 0.2 CuSO_4_·5H_2_O, 0.2 NiSO_4_·6H_2_O, 1 FeSO_4_·7H_2_O, and 5 KH_2_PO_4_. The vitamin solution V-I contained the following components (g L^−1^ deionized water): 0.04 thiamine (vitamin B1), 0.02 Ca-pantothenate (vitamin B5), 0.02 nicotinic acid (vitamin B3), and 0.008 pyridoxine (vitamin B6). The vitamin solution V-II contained the following (g L^−1^ deionized water): 0.01 biotin (vitamin B7), 0.001 cobalamin (vitamin B12), 0.1 riboflavin (vitamin B2), 0.2 pyridoxamine (vitamin B6), and 0.02 *p*-aminobenzoic acid (pABA).

**Table S1.** Concentration of total biomass $\left( x \right)$ and total lipid content in the biomass $\left( l \right)$ obtained using the medium M1 at the specified concentrations of dissolved oxygen. Mean values and standard deviations (two replicates) are shown.

| Dissolved oxygen = 20% of air saturation | | | Dissolved oxygen = 5% of air saturation | | |
| --- | --- | --- | --- | --- | --- |
| Time (h) | $x$ (g L^−1^) | $l$ (% w w^−1^) | Time (h) | $x$ (g L^−1^) | $l$ (% w w^−1^) |
| 0 | 0.3±0.0 | 27.3±1.4 | 0 | 0.5±0.0 | 26.4±1.3 |
| 6 | 1.0±0.1 | 15.3±0.8 | 10 | 1.5±0.1 | 19.5±1.0 |
| 20 | 2.1±0.1 | 16.6±0.8 | 23 | 2.4±0.1 | 19.0±0.9 |
| 30 | 2.5±0.1 | 22.7±1.1 | 30 | 2.9±0.1 | 13.3±0.7 |
| 44 | 3.1±0.2 | 23.9±1.2 | 47 | 4.1±0.2 | 13.4±0.7 |
| 55 | 3.9±0.2 | 26.8±1.3 | 57 | 4.9±0.2 | 17.9±0.9 |
| 68 | 4.1±0.2 | 28.7±1.4 | 70 | 5.8±0.3 | 19.0±0.9 |
| 77 | 3.9±0.2 | 29.3±1.5 | 82 | 6.5±0.3 | 19.0±0.9 |
| 93 | 4.3±0.2 | 31.3±1.6 | 94 | 7.1±0.4 | 18.4±0.9 |
| 101 | 4.3±0.2 | 33.0±1.6 | 105 | 7.0±0.3 | 18.9±0.9 |
| 118 | 5.0±0.2 | 33.2±1.7 | 118 | 6.7±0.3 | 19.0±0.9 |
| 126 | 5.7±0.3 | 33.2±1.7 | 129 | 6.4±0.3 | 18.3±0.9 |
| 142 | 6.2±0.3 | 40.6±2.0 | 142 | 6.3±0.3 | 18.3±0.9 |
| 149 | 6.8±0.3 | 43.2±2.2 | 153 | 6.7±0.3 | 18.3±0.9 |
|  |  |  | 168 | 6.2±0.3 | 18.9±0.9 |
|  |  |  | 169 | 6.2±0.3 | 18.9±0.9 |
|  |  |  | 175 | 6.1±0.3 | 18.5±0.9 |

**Table S2.** Concentration of total biomass $\left( x \right)$ and total lipid content in the biomass $\left( l \right)$ obtained using the medium M2 at the specified concentrations of dissolved oxygen. Mean values and standard deviations (two replicates) are shown.

| Dissolved oxygen = 20% of air saturation | | | Dissolved oxygen = 10% of air saturation | | |
| --- | --- | --- | --- | --- | --- |
| Time (h) | $x$ (g L^−1^) | $l$ (% w w^−1^) | Time (h) | $x$ (g L^−1^) | $l$ (%w w^−1^) |
| 0 | 0.4±0.0 | 29.4±1.5 | 0 | 0.5±0.0 | 29.4±1.5 |
| 12 | 0.1±0.0 | 7.4±0.4 | 12 | 0.8±0.0 | 7.8±0.4 |
| 24 | 0.5±0.0 | 8.1±0.4 | 24 | 1.4±0.1 | 7.9±0.4 |
| 36 | 1.2±0.1 | 10.4±0.5 | 36 | 1.9±0.1 | 8.6±0.4 |
| 48 | 2.3±0.1 | 18.8±0.9 | 48 | 3.3±0.2 | 9.4±0.5 |
| 60 | 3.2±0.2 | 21.5±1.1 | 60 | 4.4±0.2 | 11.8±0.6 |
| 72 | 4.3±0.2 | 19.6±1.0 | 72 | 4.9±0.2 | 13.9±0.7 |
| 84 | 4.8±0.2 | 18.7±0.9 | 84 | 4.8±0.2 | 13.4±0.7 |
| 96 | 5.9±0.3 | 18.6±0.9 | 96 | 4.9±0.2 | 12.6±0.6 |
| 108 | 6.5±0.3 | 16.7±0.8 | 108 | 4.8±0.2 | 12.5±0.6 |
| 120 | 6.9±0.3 | 15.0±0.7 | 120 | 4.7±0.2 | 12.0±0.6 |
|  |  |  | 132 | 4.7±0.2 | 9.7±0.5 |

**Table S3.** Concentration of total biomass $\left( x \right)$ and total lipid content in the biomass $\left( l \right)$ obtained using the medium M3 at the specified concentrations of dissolved oxygen. Mean values and standard deviations (two replicates) are shown.

| Dissolved oxygen = 20% of air saturation | | | Dissolved oxygen = 10% of air saturation | | |
| --- | --- | --- | --- | --- | --- |
| Time (h) | $x$ (g L^−1^) | $l$ (% w w^−1^) | Time (h) | $x$ (g L^−1^) | $l$ (% w w^−1^) |
| 0 | 1.0±0.0 | 12.2±0.6 | 0 | 0.5±0.0 | 16.1±0.8 |
| 9 | 1.1±0.1 | 13.9±0.7 | 12 | 1.1±0.1 | 14.2±0.7 |
| 22 | 2.8±0.1 | 7.4±0.4 | 22 | 2.4±0.1 | 9.0±0.5 |
| 34 | 3.4±0.2 | 11.4±0.6 | 36 | 3.7±0.2 | 11.8±0.6 |
| 46 | 4.6±0.2 | 13.1±0.7 | 46 | 4.8±0.2 | 12.6±0.6 |
| 55 | 5.0±0.3 | 13.2±0.7 | 61 | 5.3±0.3 | 17.1±0.9 |
| 70 | 5.7±0.3 | 17.8±0.9 | 70 | 5.5±0.3 | 26.1±1.3 |
| 80 | 5.7±0.3 | 28.1±1.4 | 85 | 6.4±0.3 | 35.7±1.8 |
| 95 | 6.1±0.3 | 36.6±1.8 | 94 | 6.5±0.3 | 36.7±1.8 |
| 104 | 6.7±0.3 | 40.3±2.0 | 108 | 6.9±0.3 | 37.6±1.9 |
| 119 | 6.9±0.3 | 36.2±1.8 | 117 | 6.8±0.3 | 39.6±2.0 |
| 129 | 6.8±0.3 | 32.0±1.6 | 132 | 6.5±0.3 | 42.1±2.1 |

**Table S4.** Concentration of total biomass $\left( x \right)$ and total lipid content in the biomass $\left( l \right)$ obtained using the medium M1 with the hydrolyzed lupine extract and a dissolved oxygen concentration of 20% of air saturation. Mean values and standard deviations (two replicates) are shown.

| Time (h) | $x$ (g L^−1^) | $l$ (% w w^−1^) |
| --- | --- | --- |
| 0 | 0.0±0.0 | 0.0±0.0 |
| 7 | 2.1±0.1 | 23.5±1.2 |
| 23 | 4.2±0.2 | 19.7±1.0 |
| 31 | 4.6±0.2 | 19.7±1.0 |
| 47 | 5.4±0.3 | 19.5±1.0 |
| 54 | 5.1±0.3 | 22.1±1.1 |
| 71 | 5.0±0.2 | 24.7±1.2 |
| 79 | 5.7±0.3 | 24.4±1.2 |
| 95 | 6.6±0.3 | 25.4±1.3 |
| 103 | 5.5±0.3 | 25.1±1.3 |
| 119 | 5.6±0.3 | 22.0±1.1 |
| 127 | 5.6±0.3 | 23.0±1.1 |
| 143 | 6.6±0.3 | 23.3±1.2 |
| 167 | 6.7±0.3 | 24.6±1.2 |
| 172 | 9.2±0.3 | 24.6±1.2 |

**Table S5.** Concentration of total biomass $\left( x \right)$ and total lipid content in the biomass $\left( l \right)$ obtained using the medium M1 in fed-batch operation with a dissolved oxygen concentration of 20% of air saturation. Mean values and standard deviations (two replicates) are shown.

| Time (h) | $x$ (g L^−1^) | $l$ (% w w^−1^) |
| --- | --- | --- |
| 0 | 0.7±0.0 | 29.9±1.5 |
| 20 | 1.7±0.1 | 21.2±1.1 |
| 29 | 2.5±0.1 | 14.0±0.7 |
| 44 | 3.1±0.2 | 15.4±0.8 |
| 54 | 2.9±0.1 | 14.9±0.7 |
| 68 | 3.4±0.2 | 16.0±0.8 |
| 77 | 3.6±0.2 | 14.8±0.7 |
| 92 | 3.6±0.2 | 16.3±0.8 |
| 93 | 3.8±0.2 | 17.1±0.9 |
| 101 | 6.4±0.3 | 14.0±0.7 |
| 116 | 6.8±0.3 | 15.6±0.8 |
| 142 | 7.6±0.4 | 15.6±0.8 |
| 163 | 8.3±0.4 | 17.6±0.9 |
| 164 | 7.1±0.4 | 19.7±1.0 |
| 173 | 10.7±0.5 | 19.3±1.0 |
| 187 | 12.4±0.6 | 12.9±0.6 |
| 196 | 11.2±0.6 | 19.0±0.9 |
