# Supplementary figures and images for: Production of squalene and fatty acids by Thraustochytrium sp. RT2316-16: effects of dissolved oxygen and the medium composition
Source: Bioresour Bioprocess. 2025 Sep 16;12(1):98. doi: 10.1186/s40643-025-00937-x (PMC12436261; doi:10.1186/s40643-025-00937-x)

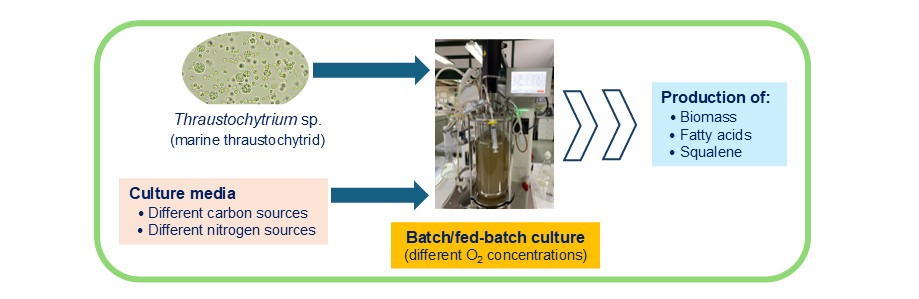

Supplement: Supplementary file 2 — Supplementary Material 2 [file 40643_2025_937_MOESM2_ESM.jpg]
